# Voluntary Hospitals

**Published:** 1922-02

**Authors:** Edgar F. Brooks

**Affiliations:** *Secretary*. The Hospitals Welfare Society for Lewisham, Deptford and Greenwich, 75, High Street, Lewisham, S.E.13.


### CORRESPONDENCE.

[Correspondence on all subjects is invited, but we cannot in any way be responsible for the opinions expressed by our correspondents, who must give their name and address as a guarantee of good faith, but not necessarily for publication. Correspondents are reminded that brevity of style and conciseness of statement greatly facilitate early insertion.]

### Voluntary Hospitals.

*To the Editor of* THE HOSPITAL AND HEALTH REVIEW.

SIR,—The question of voluntary hospitals and the means of assisting to retain the purely voluntary principle as against various schemes of a so-called provident or "contracting out" nature, is to-day of much public interest. We venture to suggest that the voluntary principle is of paramount importance for the really effective working of the hospitals, and that by an organised effort that principle can not only be maintained but greatly extended.

We have in Lewisham, Deptford and Greenwich proved this to be possible through the Hospitals Welfare Society, which has in being the Wilfred Allen Scheme (named after its founder). By means of this scheme, which is worked by over 1,700 enthusiastic voluntary workers, we are raising approximately £100 per week for local hospitals. In nine months of last year we were able to allocate £3,600, and we have every confidence that in the present year this amount will be considerably increased.

Experience has shown us that there are always a number of keen helpers only too willing and anxious to do their little for a cause which has so much to recommend it and which means alleviation of sorrow and sickness with all its resultant distress and sadness. The Wilfred Allen Scheme has been adopted with very great success in Barking, and it is well worth the attention of Hospital Management or Area Committees.

As the pioneers of the scheme, we welcome enquiries from any hospital or area, and shall always be pleased to explain matters fully and to help organise any district. Our great desire is to present this scheme as a free gift to the cause of humanity, to maintain the hospitals on the solid basis of voluntary contributions.

We feel assured that without some such practical and organised effort the hospitals must eventually become State aided, and so lose that Good Samaritan spirit which has throughout all ages been the glory of mankind in helping to "bear one another's burdens."

Yours faithfully,

EDGAR F. BROOKS, *Secretary.*

The Hospitals Welfare Society for Lewisham,
Deptford and Greenwich,
75, High Street,
Lewisham, S.E.13.

